# The intragraft vascularized bone marrow induces secondary donor-specific mystacial pad allograft tolerance

**DOI:** 10.3389/fimmu.2022.1059271

**Published:** 2022-12-12

**Authors:** Cheng-Hung Lin, Madonna Rica Anggelia, Hui-Yun Cheng, Yun-Huan Hsieh, Wen-Yu Chuang, Huang-Yu Yang, Chih-Hung Lin

**Affiliations:** ^1^ Center for Vascularized Composite Allotransplantation, Department of Plastic and Reconstructive Surgery, Chang Gung Memorial Hospital and School of Medicine, Chang Gung University, Taoyuan, Taiwan; ^2^ Department of Plastic and Reconstructive Surgery, Epworth Eastern Hospital, Melbourne, VIC, Australia; ^3^ Department of Pathology, Chang Gung Memorial Hospital, Chang Gung Medical College and Chang Gung University, Taoyuan, Taiwan; ^4^ Department of Nephrology, Chang Gung Memorial Hospital, College of Medicine, Chang Gung University, Taoyuan, Taiwan

**Keywords:** vascularized bone marrow transplantation, mystacial pad, tolerance, donor-specific, vascularized composite allotransplantation

## Abstract

**Introduction:**

Vascularized bone marrow (VBM) is essential in tolerance induction through chimerism. We hypothesized that the inclusion of VBM contributes to the induction of mystacial pad allotransplantation tolerance.

**Method:**

In this study, 19 VBM, nine mystacial pad, and six sequential VBM and mystacial pad allografts were transplanted from Brown Norway (BN) rats to Lewis (LEW) rats to test our hypothesis. The VBM recipients were divided into antilymphocyte serum (ALS) monotherapy group (two doses of ALS on day 3 pretransplantation and day 1 posttransplantation), immunosuppressant group [a week of 2 mg/kg/day tacrolimus (Tac) and 3 weeks of 3 mg/kg/day rapamycin (RPM)], and combined therapy group. The mystacial pad recipients were divided into VBM and non-VBM transplantation groups, and both groups were treated with an immunosuppression regimen that consists of ALS, Tac, and RPM. For the recipients of sequential VBM and mystacial pad allotransplantations, additional Tac was given 1 week after mystacial pad transplantation. Allograft survival, donor-specific tolerance, and chimerism level were evaluated.

**Results:**

With the administration of ALS and short-term Tac and RPM treatments, VBM recipients demonstrated long-term graft survival (>120 days) with persistent chimerism for 30 days. CD3^+^ T cells from tolerant rats showed donor-specific hyporesponsiveness and tolerance to donor skin grafts but not to third-party counterparts. Furthermore, mystacial pad graft recipients with VBM transplantation exhibited a higher allograft survival rate than those without VBM transplantation [median survival time (MST) >90 days vs. 70 days, *p* < 0.05].

**Conclusion:**

This study demonstrated that VBM transplantation is an efficient strategy to induce and maintain donor-specific tolerance for an osseous-free allograft.

## Introduction

Vascularized composite allotransplantation (VCA), an innovative field of plastic and reconstructive surgery, paves a feasible path to restore both function and esthetics for patients with large-scale tissue loss and is comparatively superior to many conventional reconstructive strategies. Face, hand, and upper extremity transplantations have been performed globally with exceptional short- to intermediate-term functional outcomes (1). However, vascularized composite allografts are composed of heterogeneous tissues with various immunogenicities. Without immunomodulation, eventual allograft rejection is inevitable (2). Immunosuppressants, such as tacrolimus (Tac) and rapamycin (RPM), are commonly used to treat rejections (3). To reduce the side effects of immunosuppressants, such as infections and malignancies, regimens of decreased immunosuppressant doses in combination with total body irradiation (TBI), lymphocyte depletion, or costimulation blockade (CoB) were suggested (4, 5).

Immune chimerism has been reported to correlate with long-term allograft tolerance. More than a decade ago, studies revealed that chimerism could be induced by a high concentration of bone marrow (BM) cell infusion (6, 7). Alternatively, chimerism could be achieved by including vascularized bone marrow (VBM) within the osseous component of the vascularized composite allograft. It is hypothesized that the microenvironment of the VBM confers an immunomodulatory effect that improves long-term graft survival (8, 9). Unlike BM transplantation, VBM transplantation does not require preconditioning with myeloablation. Therefore, graft-versus-host disease (GVHD), the overpowering donor-adaptive immunity against the recipient tissues, has yet to be reported following VBM transplantation (10).

Transplantation of a whisker-containing rat mystacial pad is a versatile VCA model (11–14). However, the lack of its bony component resulted in the need for long-term immunosuppression. A regimen with a low dose of Cyclosporine (CsA) (2 mg/kg/day) is commonly required for the entire follow-up period (200–330 days) to maintain the survival of the (modified) full face/scalp allografts from Lewis-Brown Norway (LBN) (RT11**+**n) donors onto Lewis (LEW) (RT11) recipients (15). In previous studies, osseous-containing hemiface allotransplantation allowed a tapered dose of immunosuppressant while maintaining graft survival (13, 14).

In this study, we used less of a destructive mystacial pad model with a shortened immunosuppressant regimen by performing VBM transplantation prior to mystacial pad allotransplantation. Our findings demonstrated that VBM transplantation is crucial for the induction and maintenance of long-term survival of the secondary mystacial pad allograft. With a precedent VBM transplantation, two doses of antilymphocyte serum (ALS) administration and a combination of short-term Tac and RPM conferred donor-specific tolerance to secondary mystacial pad allograft.

## Materials and methods

### Ethics statement

This study was approved by the ethics committee of the Animal Center of Linkou Chang Gung Memorial Hospital and conducted according to Institutional Animal Care and Use Committee guidelines (No. 2014121707 and 2019122015). Animal tests, including surgeries, treatments, and sample collections, were performed under the guidelines of the ethics committee of the Animal Center of Linkou Chang Gung Memorial Hospital.

### Animals

Male Brown Norway (BN) (RT1Ac), LEW (RT1Ab), and Sprague–Dawley (SD) (RT1Af) rats weighing 200–250 g that were purchased from the National Taiwan Animal Laboratory and Biolasco Ltd. served as donors, recipients, and third-party VCA donors. Animals were kept in pathogen-free conditions at the Animal Center of Linkou Chang Gung Memorial Hospital.

### Vascularized bone marrow transplantation

The donor’s right hind limb and the recipient’s left groin were used for VBM transplantations to ensure consistency and avoid variables. An approximate 2 × 3 cm^2^ superficial inferior epigastric artery (SIEA)-based elliptical skin flap was raised above the donor groin. Bipolar cautery (8 W) was used to minimize bleeding and enhance the surgical field. Once the SIEA was isolated, the animal was turned to its side to expose the entire thigh from the hip to the knee. The anterior compartment of the thigh was isolated and harvested in continuity with the femur. The knee was disarticulated first with caution once the popliteal vessels were ligated, followed by hip disarticulation. The medial thigh muscles were split using bipolar cautery while preserving the femoral artery and SIEA with care. The femoral artery was dissected as close to the inguinal ligament as possible to preserve small muscular branches and complete the flap harvested. The flap was flushed with heparinized saline and kept cold while preparing for the recipient site.

The recipient was in the supine position, and an incision was made over the inguinal crease to locate the SIEA and femoral artery. A subcutaneous pocket was made, and the SIEA was ligated with bipolar cautery. The femoral vein and artery were ligated together with one 10-0 nylon suture close to the takeoff of the SIEA. A dorsolateral window was made on the ipsilateral hind limb (16), and a broad subcutaneous tunnel was made between the inguinal incision and the dorsolateral window, allowing the passage of the surgeon’s index finger. The musculo-osseous component of the flap is buried in the inguinal pocket, and the skin paddle was inset into the dorsolateral window with nylon sutures through the subcutaneous tunnel. The suture-cuff technique described by Sucher et al. (17) was used for vessel anastomosis. Vascular orientations and tension were checked prior to the anastomoses. The venous anastomosis was performed first to reduce flap congestion, leakage, and oozing. The rat was closely observed postoperatively. Once the rat recovered with satisfactory flap observation, it was transported to the recovery unit for additional observation.

### Mystacial pad transplantation

A midline incision was made in the donor’s neck through the skin and platysma. The external jugular vein, its branches, and the posterior and anterior facial veins were isolated. The sternocleidomastoid muscle was retracted to expose the common carotid artery. It was exposed distally to the posterior belly of the digastric muscle. The posterior belly of the digastric was removed to expose the horn of the hyoid bone, and the submandibular gland was excised. The facial artery was located and preserved while branches of the common carotid arteries were ligated, including the superior thyroid, internal carotid, ascending pharyngeal, lingual, ascending palatine, and internal maxillary arteries. To expose the infraorbital nerve, the mystacial pad was partially elevated cephalically to the level of the zygoma. Once the infraorbital nerve was visualized, it was transected at the level of the periosteum. The buccal and marginal mandibular branches of the facial nerve were located anterior to the masseter. The facial nerves were dissected with masseter fascia to reduce the risk of injury. To complete the flap harvest, tendon insertion of the masseter was transected from the periosteum. The mystacial pad was islanded and contained motor nerves (buccal and marginal mandibular nerve), sensory nerves (infraorbital nerve), and the vascular pedicle (common carotid artery and external jugular vein). For the recipient, the ipsilateral mystacial pad to the donor flap was removed. The infraorbital nerve, masseter muscle, and facial nerves (buccal and marginal mandibular branches) were isolated.

Once the recipient was ready for transplantation, the flap was perfused with a heparinized lactated Ringer’s solution. The external jugular vein and common carotid artery were prepared for microvascular anastomoses using suture-cuff supermicrosurgical technique (17) at the proximal end of the common carotid artery and the external jugular vein of the recipient (River Tech Medical, TN, USA; common carotid artery and external jugular vein: inner diameter, 0.912 mm; wall thickness, 0.025 mm). Interrupted 10-0 nylon was used for end-to-end buccal and marginal mandibular nerve neurorrhaphy. Infraorbital nerve neurorrhaphy was done by using interrupted 10-0 nylon epineurial stitches. The final flap inset was completed with interrupted 6-0 nylon sutures.

### Experimental groups

**Figure 1:**
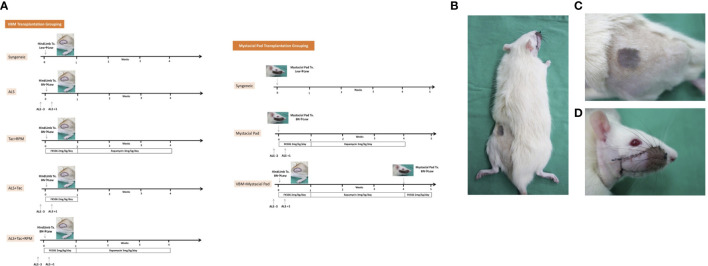
Sequential allotransplantation model of vascularized bone marrow and mystacial pad. **(A)** Grouping and timeline of immunosuppression. **(B)** Macroscopic photograph of a recipient rat with sequential transplantation. **(C)** Vascularized bone marrow allograft on 37 days post vascularized bone marrow transplantation. **(D)** Mystacial pad allograft on 7 days post mystacial pad transplantation.

VCAs were performed as follows: 1) VBM groups, a: Syngeneic VBM transplantation (Syngeneic), n = 3; b: Allogeneic transplantation administered with two doses of ALS 1 cc/rat on preoperative day 3 and on postoperative day 1 (ALS), n = 3; c: Allogeneic transplantation administered with Tac for 7 days (2 mg/kg/day) and RPM for 3 weeks (3 mg/kg/day) (Tac + RPM), n = 3; d: Allogeneic transplantation administered with two doses of ALS 1 cc/rat on preoperative day 3 and on postoperative day 1 (ALS) and Tac for 7 days (2 mg/kg/day) (ALS + Tac), n = 5; e: Allogeneic transplantation administered with two doses of ALS 1 cc/rat on preoperative day 3 and on postoperative day 1 (ALS), Tac for 7 days (2 mg/kg/day), and RPM for 3 weeks (3 mg/kg/day) (ALS + Tac + RPM), n = 5. 2) Mystacial Pad groups, a: Syngeneic mystacial pad transplantation (Syngeneic), n = 3; b: Allogeneic mystacial pad transplantation administered with two doses of ALS 1 cc/rat on preoperative day 3 and on postoperative day 1 (ALS), Tac for 7 days (2 mg/kg/day), and RPM for 3 weeks (3 mg/kg/day) (Mystacial Pad), n = 6; c: Allogeneic VBM followed by mystacial pad transplantation administered with two doses of ALS 1 cc/rat on preoperative day 3 and on postoperative day 1 (ALS), Tac for 7 days (2 mg/kg/day), and RPM for 3 weeks (3 mg/kg/day) (VBM + Mystacial Pad), n = 6 (**Figures 1A–D**).

### Peripheral blood analysis

Peripheral blood cells of VBM transplantation recipients were labeled with specific surface markers and quantitatively evaluated using the FACSCanto II flow cytometer (BD Biosciences, San Jose, CA, USA). Cell populations were analyzed per 2,000 events. Gradient forward scatter and side scatter were used to characterize lymphocytes, granulocytes, and monocytes. Two-color flow cytometry was used to detect donors (RT1Ac) and host (RT1Ab) mononuclear leukocytes (MNLs). RT1Ac^+^ granulocytes and monocytes were classified as donor-origin myeloid cells, while RT1Ac^+^ lymphocytes were classified as donor-origin lymphoid cells (**Supplementary Figure S1**). CD3, CD25, CD4, CD8, and CD161 fluorochrome-conjugated antibodies were used to detect particular T cells and natural killer (NK) cells. CD4^+^CD25^+^Foxp3^+^ cells were considered as regulatory T cells (Tregs). The gating strategies are shown in **Supplementary Figure S2**. Intracellular markers (FoxP3) were examined with permeabilization prior to antibody staining. All antibodies were ordered from BioLegend (San Diego, CA, USA) and BD Bioscience.

### Graft histology

Hind limb and mystacial pad allografts were harvested 120 days after the hind limb transplant or at the time of rejection. Tissue was fixed in 10% neutral buffered formalin (Merck KGaA, Darmstadt, Germany), embedded in paraffin (Sigma-Aldrich, St. Louis, MO, USA), sectioned with a microtome (Thermo Scientific, West Palm Beach, FL, USA), and stained with hematoxylin and eosin (H&E) (Sigma) following standard protocols.

### Secondary skin grafting

Secondary skin grafting was performed on alloVBM recipients with graft survival >90 days. Full-thickness tail skin (1 cm × 1 cm) from BN (RT1Ac; donor-matched) or SD (RT1Af; third-party) rats was transplanted onto the back of LEW (RT1Ab) recipients and secured with 6-0 Prolene sutures. Skin graft survival was observed daily by clinical inspection. Rejection manifested as erythema and complete necrosis of the skin graft.

### Magnetic cell separation

Splenocytes from naive BN (RT1Ac) and SD (RT1Af) rats were labeled with magnetic beads coated with pan T cell monoclonal antibodies (mAbs) (Miltenyi Biotech, San Diego, CA, USA), followed by negative selection on the autoMACS Pro Separator (Miltenyi) to isolate antigen-presenting non-T cells. The purity of the resultant populations was determined by flow cytometry to be higher than 95% in all experiments.

### Mixed lymphocyte reaction

Splenocytes from transplanted LEW (RT1Ab) rats labeled with Violet Proliferation Dye 450 (VPD, BD Biosciences) were cultured in mixed lymphocyte reaction medium in triplicate as previously described (10, 18). Stimulator cells [non-T cells isolated from splenocytes from naive BN (RT1Ac) and SD (RT1f) rats] were treated with 25 µg/ml mitomycin C (Sigma-Aldrich) for 10 min at 37°C under 5% CO_2_. Stimulator cells (4 × 10^5^) were cocultured with responder cells (2 × 10^5^) in 96-well U-shaped plates for 4 days at 37°C under 5% CO_2_. VPD was detected by flow cytometry. T-cell proliferation was measured by gating the CD3^+^ VPD^+^ areas.

### Statistical analysis

Kaplan–Meier method was used to analyze graft survival. The log-rank test was performed for paired comparisons. Student’s t-test was conducted for comparative analysis of two continuous independent variables. ANOVA was used to analyze more than two groups of continuous independent variables, followed by the Tukey *post-hoc* test. One-way multivariate analysis of variance (MANOVA) was done to analyze the difference between independent groups with multiple continuous independent variables. A *p*-value <0.05 was considered significant.

## Results

### A combined regimen of antilymphocyte serum with tacrolimus and rapamycin achieved long-term acceptance of vascularized bone marrow transplantation

**Figure 2:**
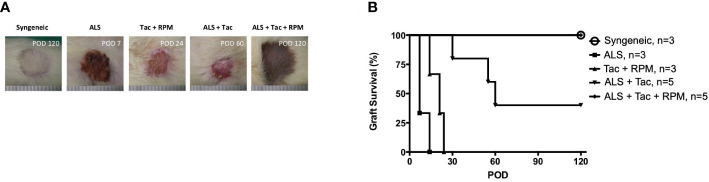
Long-term VBM transplant acceptance was achieved with antilymphocyte serum combined with tacrolimus and rapamycin. **(A)** Macroscopic photograph of allograft in each group. **(B)** Kaplan–Meier survival analysis of allograft survival of groups that received VBM allograft only. Syngeneic vs. ALS + Tac + RPM, *p* = 0.999; ALS vs. ALS + Tac + RPM, *p* = 0.005; Syngeneic vs. ALS + Tac + RPM, *p* = 0.005; ALS + Tac vs. ALS + Tac + RPM, *p* = 0.049.

The immunosuppression effect of ALS has been well established and is achieved by selective ablation of the recirculating lymphocytes and rapidly eliminating the circulating lymphocytes. In this study, using 1 cc ALS to deplete lymphocytes on preoperative day 3 and postoperative day 1 and Tac + RPM significantly prolonged allograft survival (>120 days), and the result is similar to that of the syngeneic group. However, a shorter median survival time (MST) of VBM transplants was shown in the other groups (ALS, 7; Tac + RPM, 21; ALS + Tac, 60) (**Figures 2A, B**).

### A combined regimen of antilymphocyte serum with tacrolimus and rapamycin induced peripheral multilineage chimerism in VBM transplant recipients

**Figure 3:**
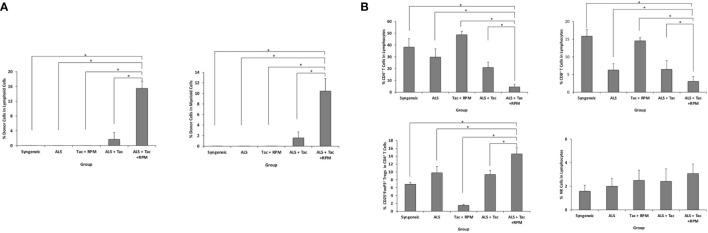
VBM transplant recipient with antilymphocyte serum combined with tacrolimus and rapamycin can induce peripheral multilineage chimerism. **(A)** Chimerism of recipients among different groups on postoperative day 30. **(B)** Peripheral blood analysis of recipients among different groups on POD 30. The asterisk denotes statistical significance (*p* < 0.05).

The hind limb comprises skin, skeletal muscles, bone, and BM. Donor-derived BM cells, especially hematopoietic stem cells, are believed to generate hematopoietic chimerism, promoting allograft tolerance. ALS and combined short-term Tac and RPM treatment could induce immune tolerance in recipients of VBM transplantation. In this group, peripheral blood multilineage chimerism was detected on postoperative day (POD) 30 (**Figure 3A**). Moreover, the recipients of this group had lower percentages of both CD8^+^ and CD4^+^ T cells and a higher level of Tregs (**Figure 3B**).

### A combined regimen of antilymphocyte serum with tacrolimus and rapamycin induced donor-specific tolerance in vascularized bone marrow transplant recipients

**Figure 4:**
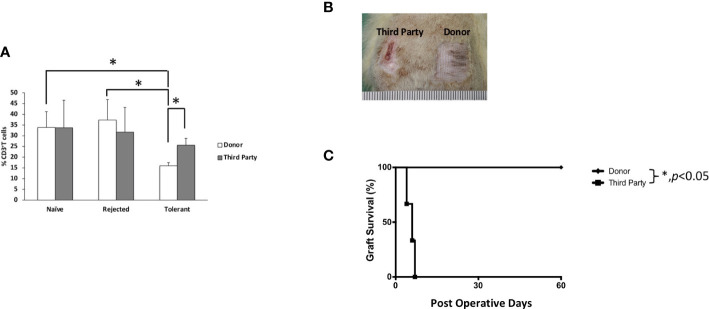
Animals treated with antilymphocyte serum, tacrolimus, and rapamycin showed T cell donor-specific hyporesponsiveness and donor-specific tolerance. **(A)** Mixed lymphocyte reaction study. **(B)** Secondary full-thickness skin allograft and Kaplan–Meier survival analysis of skin allograft survival. The asterisk denotes statistical significance (*p* < 0.05).

To test the donor-specific tolerance, secondary full-thickness skin grafts from the donor (BN) and the third-party (SD) rats were engrafted to the recipient when the VBM allograft survived for more than 90 days. All BN skin grafts survived indefinitely (>60 days), while all SD skin grafts were acutely rejected days after engraftment (MST = 6) (**Figure 4**). This suggests that the induced tolerance among VBM recipients under the ALS + Tac + RPM regimen is donor-specific.

### A critical role of vascularized hind limb transplant in tolerance induction and maintenance of secondary mystacial pad transplant

**Figure 5:**
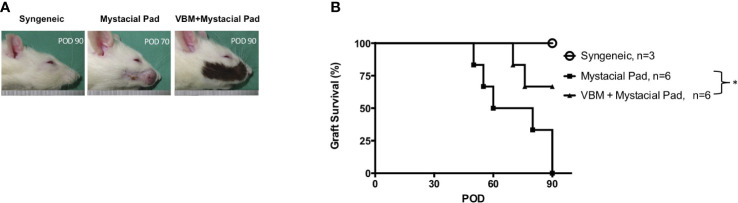
Recipients that received vascularized bone marrow transplant prior to mystacial pad transplant showed long-term allograft survival. **(A)** Macroscopic photograph of allograft in each group. **(B)** Kaplan–Meier survival analysis of allograft survival of groups that received the mystacial pad allograft. The asterisk denotes statistical significance (*p* < 0.05).

The role of vascularized hind limb transplant in the induction of tolerance to the second VCA, such as the face or mystacial pad from the same donor strain, was explored. The mystacial pad transplant was performed from BN to LEW recipients with or without precedent VBM allograft. The group that received VBM allograft 30 days prior to mystacial pad allograft showed a significantly prolonged mystacial pad allograft survival compared to the group that received mystacial pad only (>90 days vs. 65 days). The syngeneic group was used as control (**Figure 5**).

### Bone marrow in vascularized bone marrow transplant promotes tolerance *via* temporal peripheral multilineage chimerism

**Figure 6:**
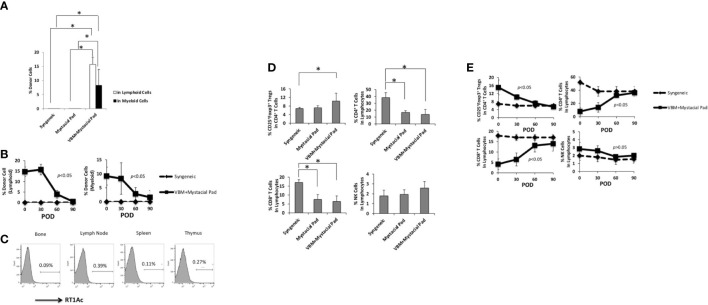
Vascularized bone marrow transplant promotes tolerance with transient peripheral multilineage chimerism. **(A)** Percentage of donor cells circulating in the recipient on 30 days post mystacial pad transplantation and **(B)** at multiple time points during observation post mystacial pad transplantation. **(C)** Chimerism was barely detected in multi-lymphoid organs in long-term tolerant animals (90 days post mystacial pad transplantation). **(D)** Peripheral blood analysis of recipients among different groups on 30 days post mystacial pad transplantation. **(E)** Peripheral blood analysis of recipients among different groups at multiple time points during observation. The asterisk denotes statistical significance (*p* < 0.05).

**Figure 3** showed that peripheral blood multilineage chimerism was detected on day 30 after VBM transplantation. Longitudinal observation displayed that the level of chimerism in the sequential allotransplantation group was decreased in 1 month after mystacial pad transplantation (**Figures 6A, B**). Furthermore, engraftment of donor cells to the bone and multilymphoid organs of these recipients was low on day 90 after mystacial pad transplantation (**Figure 6C**). No clinical signs of GVHD was detected throughout this study. According to the flow cytometry data, the circulating CD4^+^ cells, CD8^+^ T cells, Tregs, and natural killer (NK) cells were not significantly different between the recipients of mystacial pad allograft only group and those that received both VBM and mystacial pad allografts. However, the Treg level was slightly higher in the VBM and mystacial pad allograft group than those from the mystacial pad allograft only group (**Figure 6D**). Monitoring the peripheral blood in this group showed that the percentages of the studied lymphocytes were restored to the level that is similar to those of the syngeneic group (**Figure 6E**).

### Histology of secondary mystacial pad transplant showed graft acceptance

**Figure 7:**
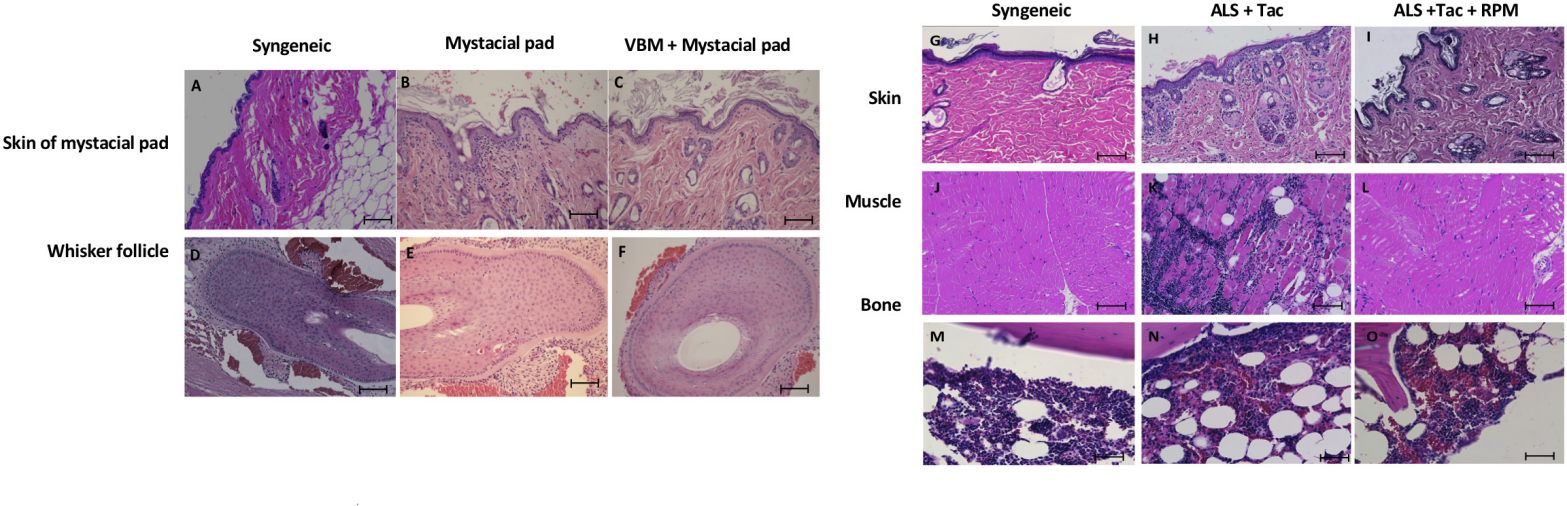
Histology of the secondary mystacial pad transplant showed graft acceptance. **(A–F)** Histopathology of the mystacial pad allograft. **(G–O)** Histopathology of the vascularized bone marrow allograft. Magnification ×100. Scale bar: 100 μm.

On day 90 after mystacial pad transplantation, we detected the whisker movements of mystacial pad allografts in the recipient that received VBM and mystacial pad allograft. Histologically, the allografts showed minimum cellular infiltration and an absence of hemorrhage (**Figures 7C, F, I, L**). Furthermore, the donor BM was viable (**Figure 7O**). Syngeneic grafts were used for comparison (**Figures 7A, D, G, J, M**). In contrast, the rejected mystacial pad revealed dense mononuclear infiltration in the epidermis, the dermis, and the whisker follicles (**Figures 7B, E**). The rejected VBM graft showed serious mononuclear infiltration in the skin and muscle, while the BM appeared viable (**Figures 7H, K, N**).

## Discussion

VCA allograft rejection and the need for lifelong nonspecific immunosuppression are significant obstacles to the broader acceptance of VCA transplantation. Our findings demonstrated that the initial VBM transplantation under an appropriate combination of ALS and short-term Tac and RPM could induce chimerism and significantly improve the survival of secondary donor-specific mystacial pad transplants. Notably, the current study presents the first successful induction of tolerance to a novel partial non-bone-containing face VCA (mystacial pad) induced and maintained by a separate vascularized hind limb transplant in a fully major histocompatibility complex mismatched rat model (BN to LEW). In addition, we achieved transplant tolerance without myeloid ablation in a more clinically relevant model.

Including a VBM transplant in the VCA model offers the benefit of reducing immunosuppressants in dosage and duration. The development of chimerism post BM transplantation correlates with long-term graft tolerance (19). Strategies and clinical trials have been developed and conducted to induce tolerance *via* immune chimerism following solid organ transplantation (SOT) (20, 21). Donor BM infusion (3.0 × 10^8^ cells/kg) showed improved cardiac and liver allograft acceptance (19, 20). However, the high dose of BM required to induce chimerism may not be feasible in the clinical setting. The transplanted VBM replenishes donor-derived hematopoietic progenitor cells and provides trophic factors to support BM replication to generate chimerism (22). Moreover, hematopoietic stem cells and stromal mesenchymal stem cells may prevent rejection (23). Our recent study demonstrated a similar efficacy of inducing donor-specific tolerance by VBM transplantation when compared to 1.5 × 10^8^ cells of BM transplantation in a mouse model (9). On the contrary, GVHD due to persistent active donor immune cells remains a significant side effect of BM transplantation (10). For instance, an unexpected report of GVHD-induced BM failure following SOT needing hematopoietic stem cell transplantation illustrated its severe morbidity and mortality in children (24).

Comparable to previous studies, our data showed a decline of chimerism in long-term follow-up while its induced long-term allograft tolerance was unaffected (18). It is suggested that transient chimerism is sufficient to induce long-term allograft tolerance. Significantly, VBM-induced transplantation tolerance was donor-specific. Supported by a previous report in the nonhuman primate model, there is an immunomodulatory role of VBM in VCA capable of reducing immunosuppressant requirements and improving results (8). No evidence to date shows a positive relationship between VBM and GVHD.

In our model, secondary mystacial pad transplantation was performed 1 month after hind limb transplantation when the chimerism level reached its peak. It differed from the previous protocols where the secondary VCA transplantation was performed >200 days (8). According to our previous studies with murine models, 1 month is a crucial period for the induction of tolerance when the chimerism level peaked (25, 26). The secondary allograft remained viable even after removing the VBM component (9). With higher antigenicity and the earlier timing of the secondary transplantation, our data showed that the lowered recipients’ immune response is less likely due to the aging process but as the significant result of VBM-induced allograft tolerance. Compared to a similar mystacial pad allograft model, we achieved tolerance with short-term immunosuppression while others prescribed long-term Tac monotherapy with reduced doses (12).

ALS efficiency varies among different batches. The previous report used ALS with a 3-day interval regimen to deplete lymphocytes for a week (27). In our model, ALS was only used for 3 days to deplete lymphocytes prior to the first allotransplantation. Therefore, combining with conventional immunosuppressant(s) is required to prolong immunosuppression. Various immunosuppressants have been trialed in VCA (28). In this study, our combined regimen of Tac and RPM worked well to support ALS in promoting graft tolerance.

Applications of Tac in animal face VCA studies have been described previously. Tac enhances nerve regeneration and promotes functional recovery of VCA (12, 29). On the other hand, RPM has potent immunosuppressive and anti-inflammatory properties (30) that selectively preserve CD4^+^CD25^+^Foxp3^+^ Tregs and inhibit effector T-cell expansion (31, 32). RPM has also been reported to promote Treg expansion (33, 34). It facilitates robust and rapid Treg proliferation *via* T cell receptor (TCR) and cluster of differentiation 28 (CD28) or interleukin (IL)-2 (32, 35). Instead of blocking phosphatidylinositol 3-kinases (PI3K)/Ak strain transforming (Akt)/mammalian target of rapamycin (mTOR) pathway in effector T cells, RPM upregulates the expression of the antiapoptotic bcl-2 family members in Tregs (36). RPM could also enhance Foxp3 expression, and this effect is endogenous transforming growth factor (TGF)-β-dependent (37). In this study, RPM may help to maintain low alloantigen-triggered CD4^+^ and CD8^+^ T cell proliferation and support rapid CD4^+^CD25^+^Foxp3^+^ Treg recovery in the crucial time point after ALS-induced lymphocyte depletion and VBM transplantation, thus leading to eventual allograft tolerance. Maintaining long-term CD4^+^ and CD8^+^ T-cell suppression is crucial for graft acceptance (9). Comparing Treg and CD4^+^ and CD8^+^ T cells in the ALS group and Tac and RPM group, the high Treg level in both groups showed no significant difference, whereas the CD4^+^ and CD8^+^ T cell levels in the ALS group appeared to be higher. The data supported the importance of the Treg ratio to effector T cell and highlighted the importance of CD4^+^ and CD8^+^ T cells in allograft tolerance induction (38).

Tregs play a crucial role for the induction of immune tolerance and have potential application as cell-based therapy in transplantation. Fisher et al. (39) showed that infusion of induced Tregs promoted donor-specific tolerance in the hind limb VCA model. However, Treg administration alone may not be sufficient to prolong allograft survival in the long-term. Additional treatments such as T-cell depletion, immunosuppressant, and IL therapy were also needed to improve its efficacy (40–42).

Although the migration of donor femoral BM cells to recipients’ immune organs such as the thymus was not detected in this model, donor-specific tolerance was observed. It was reported that central tolerance occurs at sites where T-cell education takes place *via* positive and negative selections (8, 38). More specifically, our previous study proved that less donor-reactive Vβ5-expressing CD4^+^ T cells were detected in tolerant recipients, hence suggesting the role of thymus in central tolerance (38). Moreover, donor-specific chimerism was diminished in VCA recipients with thymectomy (43–45). The pilot data regarding engineered donor–recipient hybrid thymus were reported have potential to promote a dominant regulation of alloreactivity to achieve allograft tolerance (46).

Finally, the success in prolonging mystacial pad allograft survival may be advantageous for advancing VCA studies. Orthotopic mystacial pad transplantation is a unique model for nerve regeneration studies after allotransplantation (47, 48). Long-term allograft survival may facilitate the longitudinal study of functional outcomes, which is crucial in VCA studies.

## Conclusion

In this study, we established a reliable and reproducible strategy to induce mystacial pad allograft tolerance in the rat. The findings not only open new perspectives for the crucial role of VBM transplant in the induction and maintenance of VCA tolerance but also offer an effective approach to induce tolerance of the secondary allograft with only short-term immunosuppression.

## Data availability statement

The original contributions presented in the study are included in the article/**Supplementary Material**. Further inquiries can be directed to the corresponding authors.

## Ethics statement

The animal study was reviewed and approved by the Ethics Committee of the Animal Center of Linkou Chang Gung Memorial Hospital (Ethic approval file No. 2014121707 and 2019122015).

## Author contributions

Che-HL and Chi-HL conceived and designed the study. Che-HL and MA performed data acquisition. Che-HL, MA, H-YC, W-YC and H-YY analyzed and interpreted the data. Che-HL, MA, H-YC, Y-HH wrote the manuscript. Che-HL and Chi-HL reviewed the final writing. All authors contributed to the article and approved the submitted version.
