# Supplementary figures and images for: The intragraft vascularized bone marrow induces secondary donor-specific mystacial pad allograft tolerance
Source: Front Immunol. 2022 Dec 12;13:1059271. doi: 10.3389/fimmu.2022.1059271 (PMC9791084; doi:10.3389/fimmu.2022.1059271)

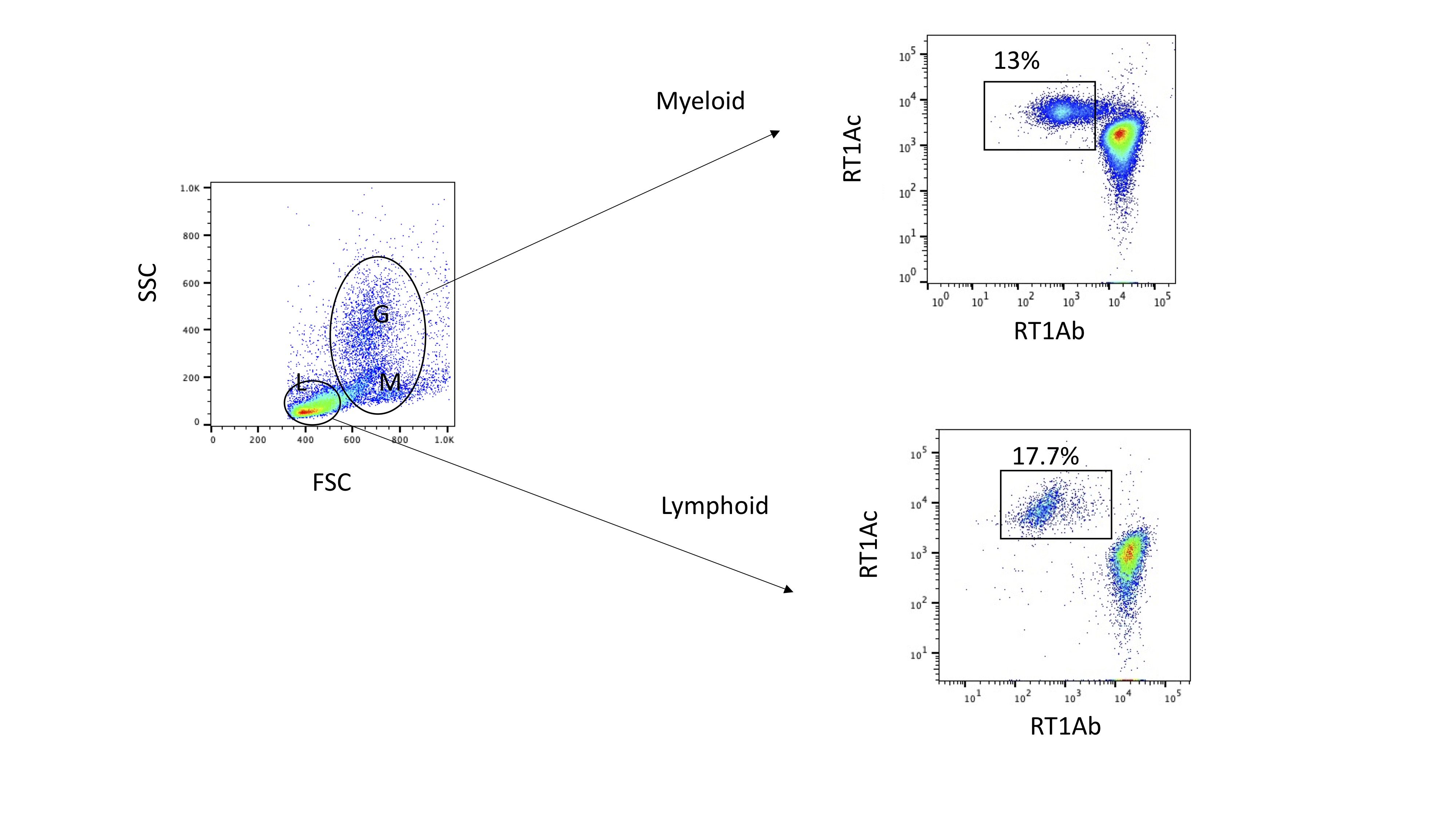

Supplement: Supplementary file 1 [file Image_1.jpeg]

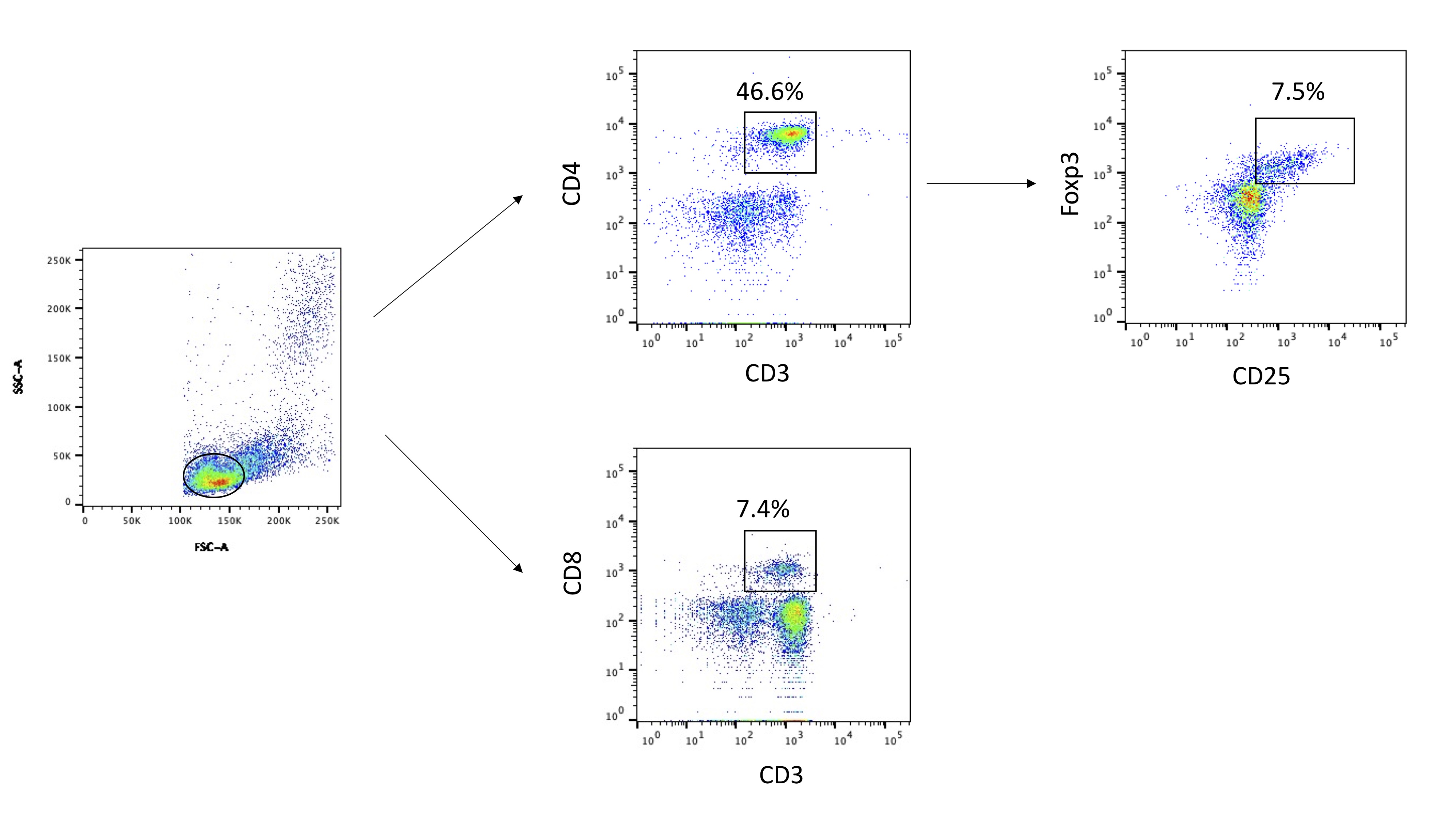

Supplement: Supplementary file 2 [file Image_2.jpeg]

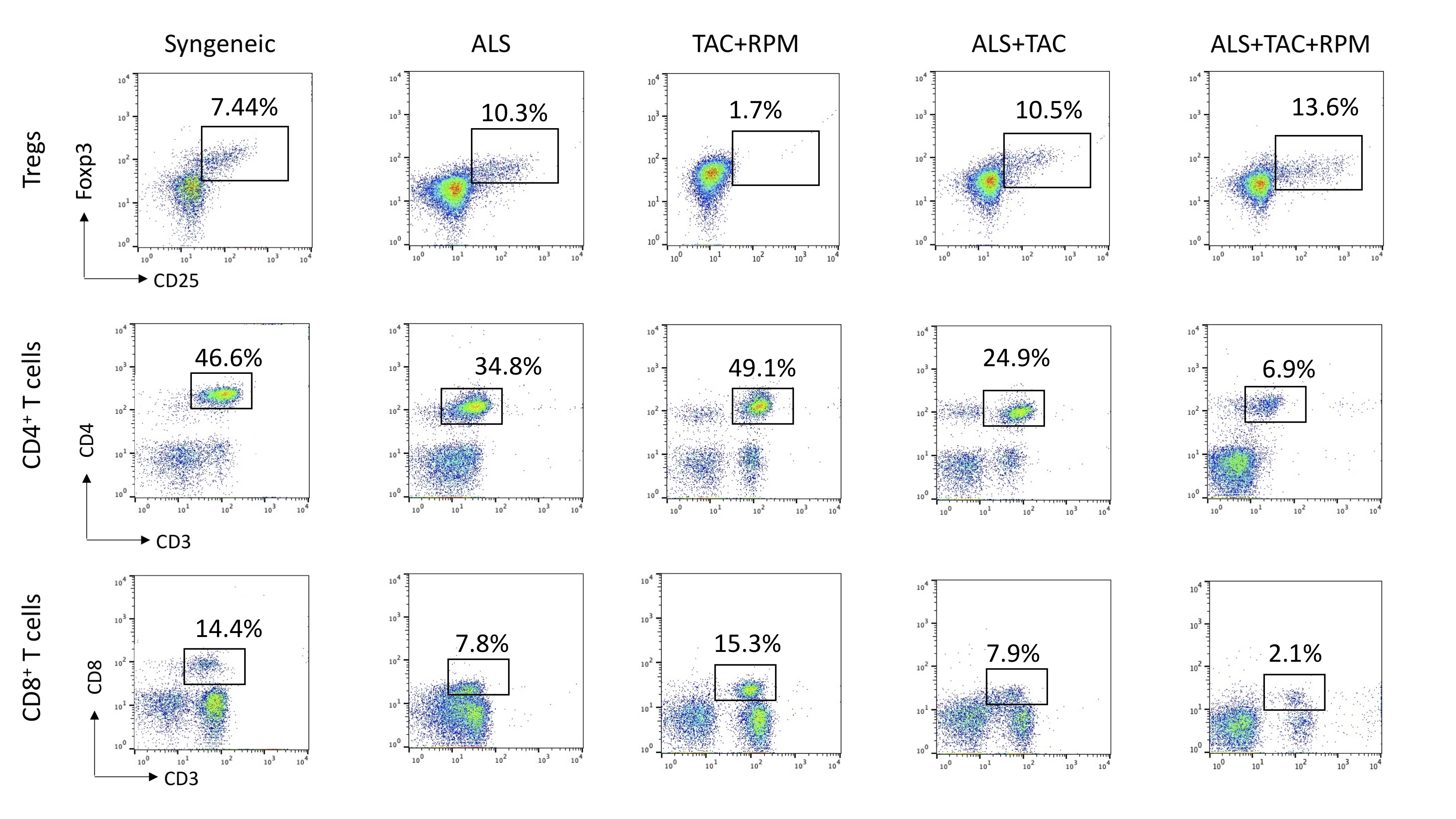

Supplement: Supplementary file 3 [file Image_3.jpeg]
